# Tailored efficient energy transfer Tb^3+^, Eu^3+^ activated/co-activated LiAl(PO_3_)_4_ phosphor by substitution of alkali metals: the effect of charge compensation

**DOI:** 10.1039/d3ra03115b

**Published:** 2023-09-01

**Authors:** Prashant N. Parale, Abhijeet R. Kadam, S. J. Dhoble, K. V. Dabre

**Affiliations:** a Department of Physics, R. T. M. Nagpur University Nagpur – 440033 India; b Department of Physics, Taywade College Koradi Nagpur – 441111 India; c Department of Physics, School of Basic and Applied Sciences, MGM University Chhatrapati Sambhajinagar Aurangabad – 431003 India arkadam6@gmail.com

## Abstract

Phosphites are the new emerging candidates in the field of luminescence in the modern era. In the present investigation, Tb^3+^/Eu^3+^ activated/co-activated LiAl(PO_3_)_4_ phosphor was prepared by a wet chemical method, and the effect of R^+^ (Na^+^, K^+^) ions on photoluminescence (PL) properties of these phosphors are investigated. Phase identification and crystal structure of the prepared phosphor were determined using XRD and Rietveld refinement, respectively. Morphological study and elemental analysis of the proposed phosphor with elemental analysis of the sample were performed using SEM and EDS. The PL properties of the proposed phosphor showed three simultaneous emission peaks in the visible range, giving color-tunable emission. The charge compensation of Na^+^ and K^+^ ions make a significant impact on the PL intensity of Tb^3+^, Eu^3+^ co-activated LiAl(PO_3_)_4_ phosphors. The PL intensity of Tb^3+^, Eu^3+^ co-activated LiAl(PO_3_)_4_ phosphors was significantly enhanced by factors 1.2 and 1.4 when Na^+^ and K^+^ charge compensators, respectively, were introduced. To manifest the charge compensation effect of alkali metals the optimum intense sample in the co-doped sample was used. These results indicate the potential candidacy of the studied phosphor for further improvement in PL properties for application in solid-state lighting.

## Abstract 2

The studied phosphor LiAl(PO_3_)_4_: *x* mol% Tb^3+^, *y* mol% Eu^3+^, *z* mol% R^+^ (R = Na, K) shows excellent photoluminescence properties with efficient energy transfer from Tb^3+^ to Eu^3+^, enhancement in intensity using charge compensation and show the photochromic nature.

## Graphical abstract

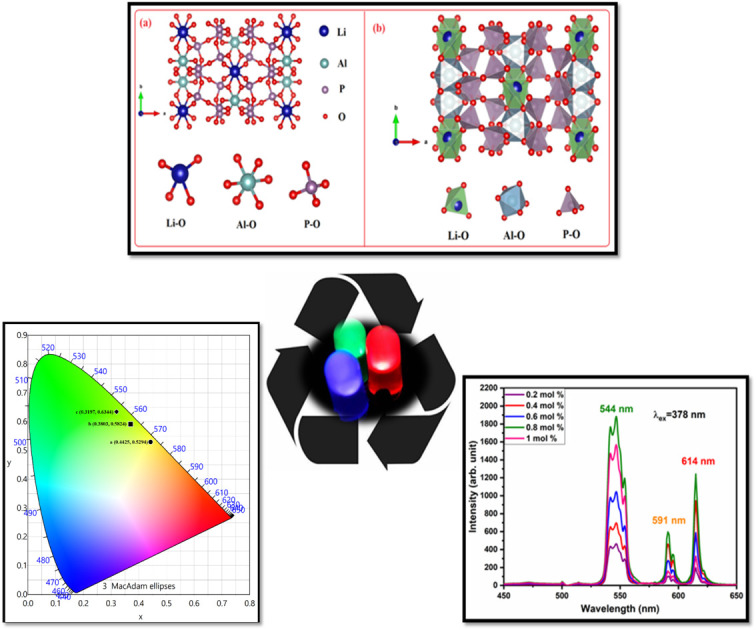

## 1. Introduction

The scientific world has been paying close attention to lanthanide-based phosphors because of their outstanding photophysical characteristics,^1^ including narrow emission,^2^ significant Stokes shift,^3^ strong photostability,^4,5^ photochromic luminescence,^6,7^ and long lifespan.^5,8,9^ They are employed in almost all areas of science and technology, including solid-state lighting,^10–13^ scintillators,^14,15^ photonics,^16–18^ detectors,^19,20^ catalytic processes,^21,22^ vitality,^23,24^ and environmentalism,^25^ due to their exceptional photophysical properties. The lanthanides Dy^3+^, Tb^3+^, and Eu^3+^ ions exhibit extremely different emission bands in the visible and NUV regions, making them a desirable contender for WLEDs and solar power.^26–33^ Nevertheless, the performance of the doping and the kind of inorganic host in which it was successfully doped determine the luminescence performance, longevity, and quantum yield.^34,35^ The inorganic oxides, phosphates, and fluoride phosphors have a number of advantageous characteristics that make them suitable luminescence hosts. The simplicity of the synthesis, lack of toxicities, high level of structural, chemical, and mechanical integrity, diversity of structure, photochromic optical characteristics and endurance, and self-activated luminescence are a few of the characteristics.^36–40^ The host-dopant energy transfer exerts considerable control over the luminescence lifespan and quantum efficiency of systems that are doped with lanthanides to produce luminous hosts.^41,42^ The choice of dopant and host has been shown in multiple papers from all over the world on Ln^3+^-activated phosphates to have a very large impact on the host-to-dopant energy transfer efficiency. Overall, when the size of the dopant can be accommodated inside the host lattice, the host-to-dopant exchange of energy is enhanced. A number of additional difficulties in producing luminescence hosts with the best optical properties are the strain and defect development caused by the size and charge imbalance among rare earth ions such as lanthanides and the host matrices.

A monovalent alkali metal ion co-doped as a charge compensator is considered the most popular solution to this problem.^43,44^ The generation of cationic vacancies, which could offer non-radiative pathways and lower the light output of such phosphors, is thought to result from trivalent doping at a divalent site.^45–47^ Lithium, sodium, and potassium are the most commonly used alkali metals. To observe the impact of the charge compensation effect, many rare earth-activated luminous phosphors are currently being researched. Sobierajska *et al.*^48^ examined the impact of substituting lithium as a charge compensator on the luminescence properties of Ca_10_(PO_4_)_6_F_2_:Eu^3+^ phosphor. In order to look into the site occupancy choice for the charge compensation co-doping, the concentration of Eu^3+^ ions was 1 mol% and the concentration of Li^+^ ions was between 0.5 and 5 mol%. According to the luminescence characteristics of Eu^3+^ ions, the Ca1 site is predominantly substituted for Ca2 when the co-dopant (Li^+^ ions) concentration is below 2 mol%. The inclusion of Eu^3+^ ions at the Ca2 site was indicated by a red shift in the C-T band when the co-dopant level increased. Cao *et al.*^49^ disclosed that the solid-state chemical technique was used to create the CaZrO_3_:Eu^3+^, Bi^3+^, and Li^+^ phosphors. The CaZrO_3_:Eu^3+^, Bi^3+^ phosphor's clearly luminescent qualities can be improved by 1.6 times by co-doping Li^+^ ions in their fluxing and charge-compensating roles. Yang *et al.*^50^ used Ba_0.92_SiO_3_:0.08Eu, Ba_0.88_SiO_3_:0.08Eu, and Ba_0.84_SiO_3_:0.08Eu to study the effect and mechanism of varied charge compensation on the luminous properties of Eu-doped BaSiO_3_. 0.08R^+^ (R = Na, K) phosphors were prepared using the technique of co-precipitation, and the precursors used were calcined in air. It was discovered that the phosphor Ba_0.84_SiO_3_:0.08Eu, 0.08Na^+^ performed more effectively at high temperatures and had an extended lifespan than the phosphor Ba_0.92_SiO_3_:0.08Eu, indicating that the co-doping of Na^+^ ions was advantageous to this phosphor. Various kinds of hosts have nevertheless currently been studied. For instance, the charge compensation for phosphite-based phosphors has not been the subject of any known studies. Thus, in the present study, the effect of charge compensation of alkali (Na^+^, K^+^) on the photoluminescence (PL) properties of Tb^3+^, Eu^3+^ activated/co-activated LiAl(PO_3_)_4_ phosphite phosphors was investigated.

## 2. Experimental

Pure and Tb^3+^, Eu^3+^ activated/co-activated samples of LiAl(PO_3_)_4_ phosphors were synthesized by the wet chemical method.^51^ In the proposed synthesis, LiNO_3_ 99% extra pure, Al(NO_3_)_3_ 98% extra pure, NH_4_H_2_PO_4_ 98% extra pure, NaNO_3_ 99% extra pure, KNO_3_ 99% extra pure, Eu_2_O_3_ 99.9% AR grade, Tb_4_O_7_ 99.9% AR grade were used. The typical chemical reaction for the synthesis of the host material is given as follows:LiNO_3_ + 4(NH_4_H_2_PO_4_) + Al(NO_3_)_3_ → LiAl(PO_3_)_4_ + 4(H_2_O) + 4(NH_3_) + 2H_2_↑

A predefined amount of all reagents was taken in a stoichiometric ratio for the preparation of the host material. These reagents were dissolved in a separate beaker containing around 50 ml of double distilled water using a magnetic stirrer, and all these mixtures were mixed together in one beaker. The final solution was continuously stirred for 45 min with constant heating for proper mixing and to evaporate the excess water. A white precipitate was obtained, which was kept in the hot air oven for 12 hours at 90 °C. After that samples were cooled at room temperature and the final product was obtained. For the synthesis of Tb^3+^, Eu^3+^ activated/co-activated LiAl(PO_3_)_4_ phosphite phosphors, stoichiometric amounts of Tb_2_O_3_ and Eu_2_O_3_ (aiming to replace Li^+^ ion) were dissolved in nitric acid and then added in the solution mixture and rest of the procedure was kept the same. A similar procedure was adopted to prepare alkali (Na^+^, K^+^) metal ion-doped samples. In singly activated phosphor the concentrations of Tb^3+^ and Eu^3+^ ions were varied from 0.3 to 1.5 mol%. In the co-activated phosphor, the concentration of Tb^3+^ ions was kept fixed at 1 mol% and the concentration of Eu^3+^ ion was varied from 0.3 to 1.5 mol%. While studying the charge compensation effect in co-activated phosphor the concentrations of Tb^3+^ and Eu^3+^ ions were fixed at 1 mol% and 1.5 mol%, respectively, and the concentration of alkali (Na^+^/K^+^) was varied from 0.2 to 1 mol%. The final products obtained were then used as is for further characterization.

X-ray diffraction (XRD) analysis of phosphors was carried out to clarify the phases and crystalline structure using Rigaku miniflex d 600 X-ray diffractometer with Cu Kα radiation (*λ* = 0.154059 nm) operated at 40 kV, 15 mA, and the patterns were recorded in the range of 10–90°. The infrared spectra were recorded on an alpha II Brucker Fourier transform infrared (FTIR) spectrometer. Morphological and elemental mapping analyses were performed using a scanning electron microscope (Carl Zeiss EVO-18) equipped with energy-dispersive X-ray spectroscopy (EDS). The PL excitation and emission spectra of phosphor samples were recorded using a SHIMADZU spectrofluorophotometer RF-5301 PC.

## 3. Results and discussion

### 3.1 XRD analysis and crystal structure

**Fig. 1:**
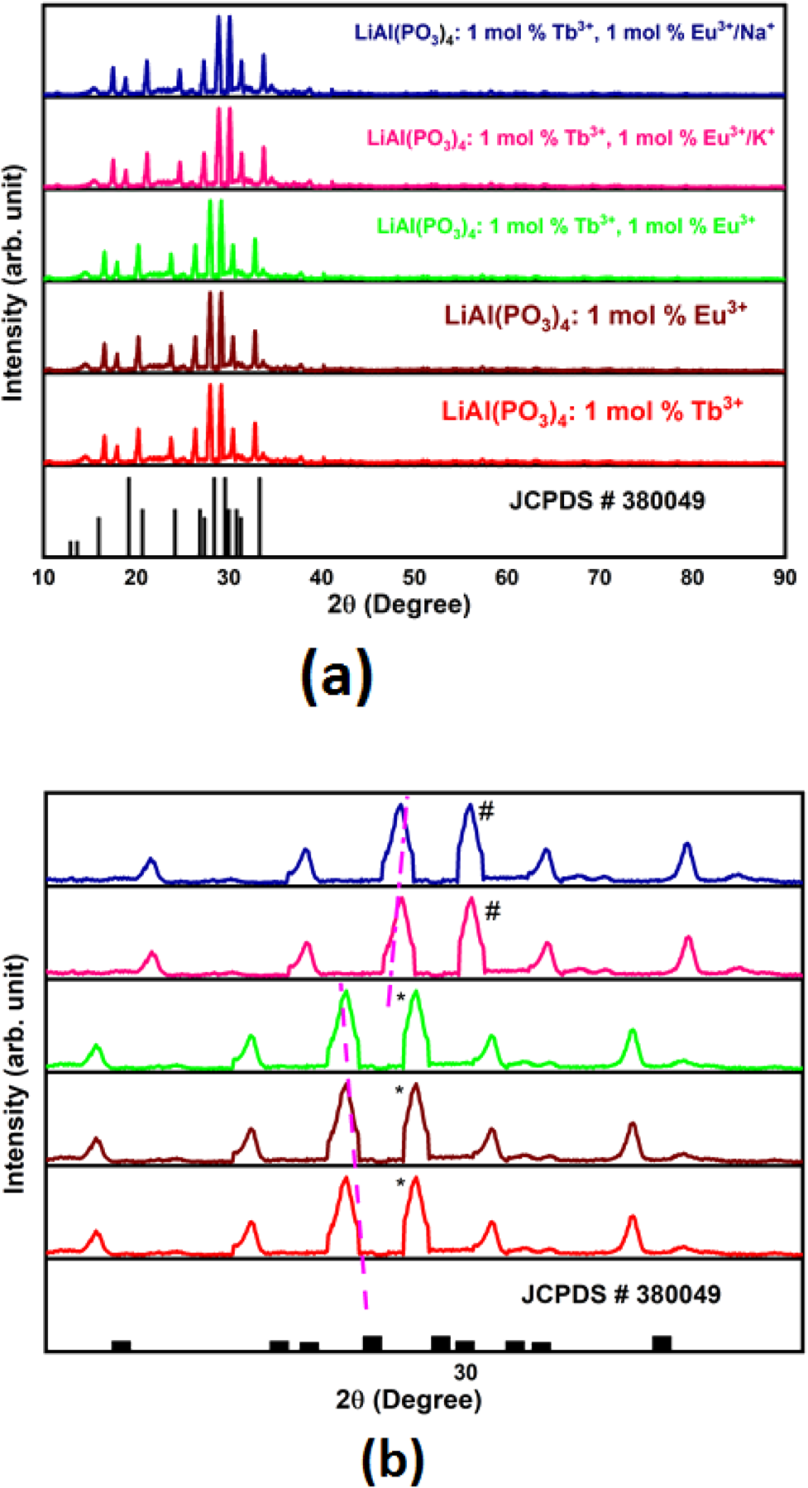
(a) XRD patterns of Tb^3+^/Eu^3+^ activated/co-activated LiAl(PO_3_)_4_ phosphors and LiAl(PO_3_)_4_ phosphors doped with Tb^3+^/Eu^3+^/M^1+^ (M = Na, K) compared with the standard XRD. (b) Zoomed XRD patterns showing shifting of the diffraction peaks.

Powder XRD patterns of Tb^3+^/Eu^3+^-activated/co-activated LiAl(PO_3_)_4_ and LiAl(PO_3_)_4_ phosphors doped with Tb^3+^/Eu^3+^/M^1+^ (M = Na, K) are depicted in Fig. 1(a). As observed in this figure, the XRDs of all prepared phosphors are in good agreement with the standard pattern, JCPDS PDF no. #380049. However, some peaks are shifting towards a lower angle [Fig. 1(b)]. The shift in the XRD peaks towards lower angles might be due to change in the ionic radii in host lattice and rare earth ions and alkali ions doped (Tb^3+^, *r* = 1.04 Å CN = 8; Eu^3+^, *r* = 1.066 Å, CN = 8), (Li^+^, *r* = 0.92 Å CN = 6, Na^1+^, *r* = 1.18 Å CN = 8; K^1+^, *r* = 1.51 Å CN = 8). Moreover, there were no extra impurity peaks, and hence, the samples were prepared in a very good manner.

**Fig. 2:**
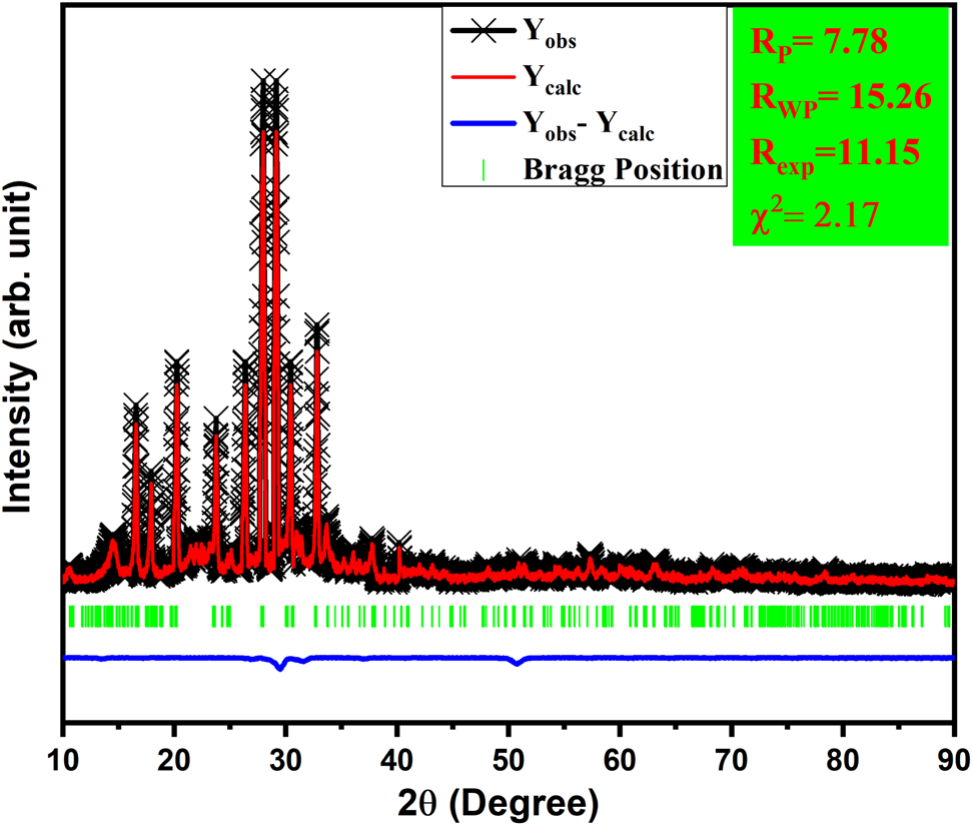
Rietveld refinement of LiAl(PO_3_)_4_ phosphor doped with Tb^3+^ (1 mol%).

To validate this statement, Rietveld refinement was performed on the XRD pattern, as shown in Fig. 2. Rietveld refinement was carried out together with their difference profile and Bragg locations utilizing the Fullprof Suite programme to verify this claim.^52–54^ The visualization requirements were met by the reliability factors along with the parameters *R*_wp_ = 15.26, *R*_p_ = 7.78, *R*_exp_ = 11.15, and *χ*^2^ = 2.17. The volume of the unit cell obtained from the Rietveld refinement was 916.439 Å^3^.

**Fig. 3:**
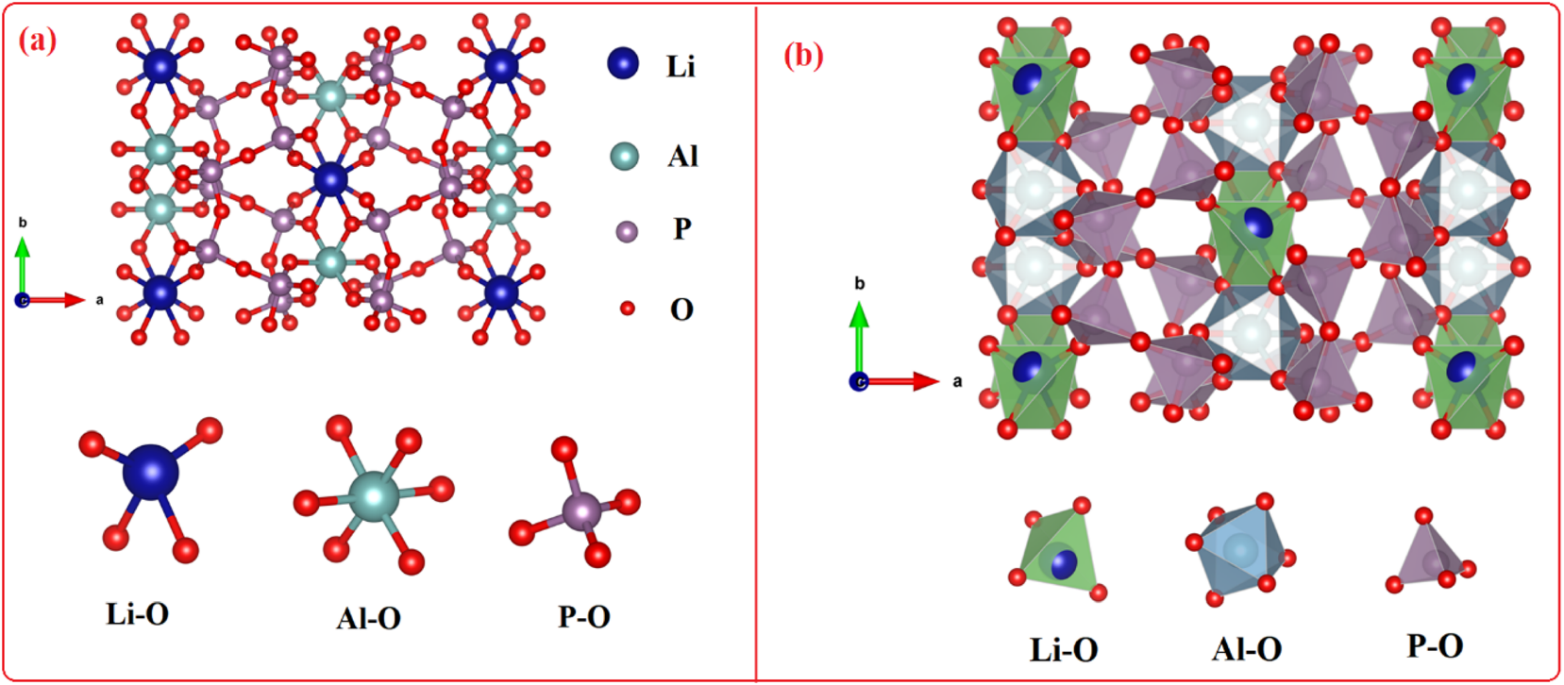
The crystal structure of LiAl(PO_3_)_4_ material obtained from Rietveld refinement.

Fig. 3 depicts the ball and stick and the polyhedral crystal structure orthorhombic with lattice data *a* = 12.43 Å, *b* = 8.22 Å, *c* = 8.91 Å, and *α* = *β* = *λ* = 90.00°. *Pbcn* space group is the crystallization space for LiAl(PO_3_)_4_. Li^+^ forms deformed LiO_4_ tetrahedra by bonding with four O^2−^ atoms, which share corners with four comparable PO_4_ tetrahedra and an edge–edge with an AlO_6_ octahedron. There are four Li–O bond lengths in total: two shorter (1.92 Å) and two longer (2.07 Å). Al^3+^ forms AlO_6_ octahedra by bonding with six O^2−^ atoms, which share corners with six PO_4_ tetrahedra and an edge–edge with one LiO_4_ tetrahedra. Al–O bond separations vary and range from 1.89 Å to 1.92 Å. Two different P^5+^ locations exist. In the first P^5+^ site, P^5+^ forms PO_4_ tetrahedra by bonding with four O^2−^ atoms. These tetrahedra share corners with one AlO_6_ octahedron, two equivalent LiO_4_ tetrahedra, and two equivalent PO_4_ tetrahedra. The octahedral corner-sharing tilt angles are 42°. The P–O bond separations vary and range from 1.48 Å to 1.62 Å. In the second P^5+^ site, P^5+^ forms PO_4_ tetrahedra that share corners with two equivalent AlO_6_ octahedra and corners with two equivalent PO_4_ tetrahedra *via* bonding with four O^2−^ atoms. The octahedral corner-sharing tilt angles range from 42° to 47°. There is a range of P–O bond separations between 1.50 Å and 1.60 Å. There are six incompatible O^2−^ locations. O^2−^ is coupled to two P^5+^ atoms at the first O^2−^ site in a bent 120° geometry that is deformed. O^2−^ is bound to two P^5+^ atoms in the second O^2−^ site at an abnormal angle of 120°. One Al^3+^ and one P^5+^ atoms are connected by a 150° bent, twisted geometry to O^2−^ at the third O^2−^ site. O^2−^ is bound to one Li^+^ and one P^5+^ atom in a bent 150° geometry at the fourth O^2−^ location. O^2−^ is bound to one Al^3+^ and one P^5+^ atom in bent 120° distortion geometry at the fifth O^2−^ site. One Li^+^, one Al^3+^, and one P^5+^ atom are joined by O^2−^ in a deformed trigonal planar geometry at the sixth O^2−^ site. The structural parameters for LiAl(PO_3_)_4_ phosphor are summarized in Table 1.

**Table 1:** Overview of the structural parameters of the LiAl(PO_3_)_4_ material

		*x*	*y*	*z*	Occ.	U	Site	Sym.
1	O1	0.2442	0.1049	0.0804	1	0.006	8d	1
2	O2	0.3951	0.4079	0.3732	1	0.006	8d	1
3	O3	0.3199	0.1413	0.4604	1	0.006	8d	1
4	O4	0.1204	0.3658	0.3773	1	0.006	8d	1
5	O5	0.0623	0.1985	0.1353	1	0.006	8d	1
6	O6	0.4329	0.0286	0.1344	1	0.006	8d	1
7	P1	0.13919	0.18251	0.00584	1	0.006	8d	1
8	P2	0.35134	0.03648	0.01166	1	0.006	8d	1
9	Li1	0	0.0028	0.25	1	0.006	4c	2
10	Al1	0	0.3676	0.25	1	0.006	4c	2

### 3.2 SEM and EDS analysis

**Fig. 4:**
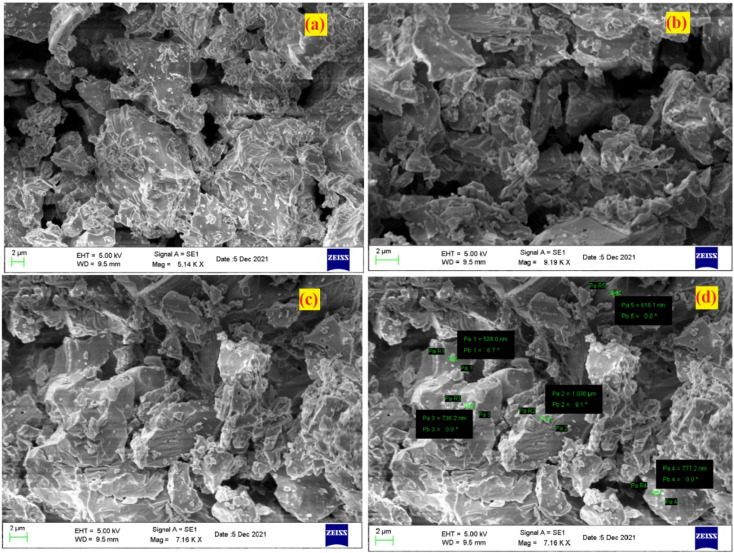
SEM micrograph of LiAl(PO_3_)_4_phosphors doped with Tb^3+^/Eu^3+^/M^1+^ (M = Na, K).

In order to investigate the morphology and elemental mapping in the prepared phosphors, SEM and EDS analysis were performed. SEM micrographs of the prepared phosphors are shown in Fig. 4. As observed in Fig. 4, samples are in the micrometer range with irregular shapes and sizes. The microscopic size of the prepared phosphor makes it a potential phosphor candidate as per the point of view of WLED.^53,55,56^

**Fig. 5:**
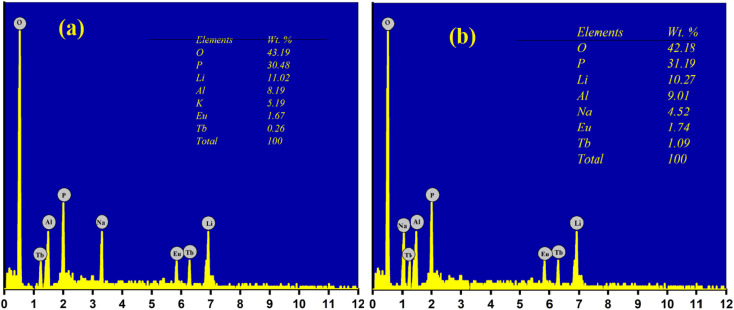
EDS analysis of LiAl(PO_3_)_4_ phosphors doped with Tb^3+^/Eu^3+^/M^1+^ [M = Na (a), K (b)].

EDS analysis was performed in order to investigate the presence of elements or any other impurities in the prepared phosphor. The EDS analysis of LiAl(PO_3_)_4_ phosphors doped with Tb^3+^/Eu^3+^/M^1+^ (M = Na, K) is depicted in Fig. 5. EDS confirmed that all the raw materials used in the synthesis were present in the prepared phosphor, and no other impurities were observed. The insets of Fig. 5(a) and (b) show the wt% distribution of precursors used in the prepared phosphor.

### 3.3 Photoluminescence investigation

**Fig. 6:**
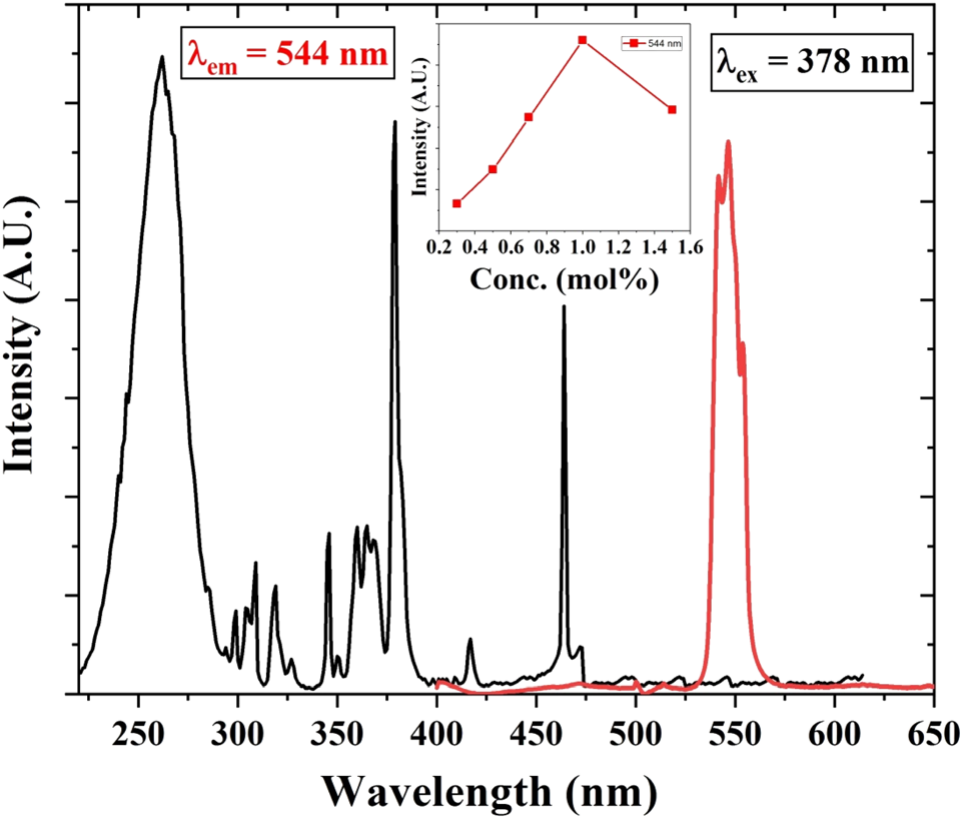
PL excitation and emission spectra of Tb^3+^ (1 mol%) activated LiAl(PO_3_)_4_ phosphors (inset: the variation of emission intensity of 544 nm peak with Tb^3+^ ion concentration).

The PL excitation and emission spectra of Tb^3+^ (1 mol%) activated LiAl(PO_3_)_4_ phosphor are depicted in Fig. 6. The excitation spectra of the 544 nm emission of phosphor exhibit a broad excitation band in the UV region centred at 262 nm and characteristic peaks in near UV and blue regions. These characteristic excitation peaks attributed to f–f transitions ^7^F_6_ → ^5^D_2_, ^7^F_6_ → ^5^L_10_, and ^7^F_6_ → ^5^D_3_ are positioned at 345 nm, 365 nm, and 378 nm, respectively.^57–59^ The PL emission spectra of Tb^3+^-activated LiAl(PO_3_)_4_ triggered by excitation at 378 nm showed one dominant emission peak in the green region positioned at 544 nm. The peak attributed at 544 nm is due to the ^5^D_4_ → ^7^F_5_ transition.^60^ It was also observed that the emission intensity increased with Tb^3+^ ion concentration up to 1 mol%, after which the concentration quenching was observed.

**Fig. 7:**
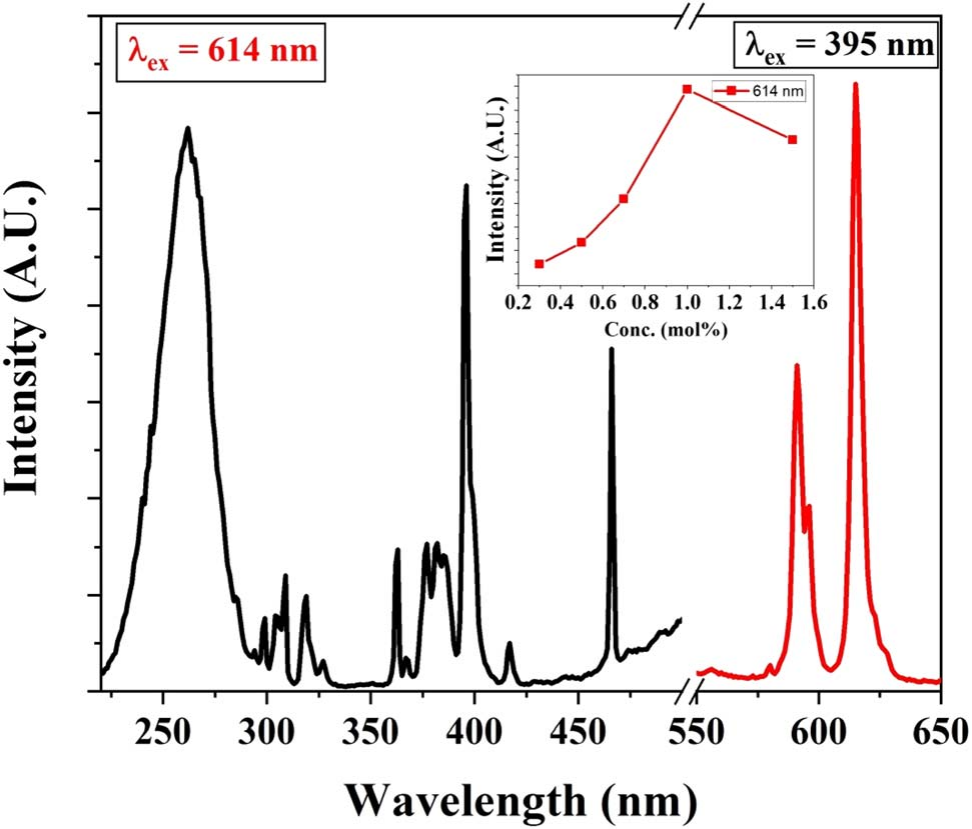
PL excitation and emission spectra of Eu^3+^ (1 mol%) activated LiAl(PO_3_)_4_ phosphors (inset: the variation of emission intensity of 544 nm peak with Tb^3+^ ion concentration).

Similarly, Eu^3+^ ion-activated LiAl(PO_3_)_4_ phosphors were also investigated by PL spectroscopy. The PL excitation and emission spectra of the Eu^3+^ activated LiAl(PO_3_)_4_ phosphors are displayed in Fig. 7. The PL excitation spectra monitored at 614 nm exhibited 4 characteristic excitation bands positioned at 261 nm, 395 nm, 465 nm, and 535 nm. The peak observed at 261 nm is due to the O^2−^ → Eu^3+^ charge transfer band. However, other bands observed in the NUV region (395 nm), blue region (465 nm), and green region (535 nm) are due to ^7^F_0_ → ^5^L_6_, ^7^F_0_ → ^5^D_3_ and ^7^F_0_ → ^5^D_1_ transition, respectively.^61,62^

Eu^3+^-activated LiAl(PO_3_)_4_ phosphors after further excitation at 395 nm gave two characteristic emission peaks positioned at 591 nm and 614 nm in the orange and red regions, which are ascribed to ^5^D_0_ → ^7^F_1_ magnetic dipole transition and ^5^D_0_ → ^7^F_2_ electric dipole transition of Eu^3+^ ions, respectively.^32,63,64^ The peak due to the electric dipole transition is more intense than the magnetic dipole transition, which indicates that the location of the Eu^3+^ ion is at the non-centrosymmetric site in the host lattice. The variation in the emission intensity of the Eu^3+^-activated LiAl(PO_3_)_4_ phosphors showed a pattern similar to that of Tb^3+^-activated LiAl(PO_3_)_4_ phosphors, which indicated that the doping of rare earth ion occurred at the same site in the host lattices.

The reason behind the concentration quenching effect observed in the PL emission intensity of the rare earth ion-activated LiAl(PO_3_)_4_ phosphor is the critical transfer distance (*R*_C_), which is the minimum distance between the nearest activator at a critical concentration *X*_C_. The critical transfer distance can be calculated using Blasse formula;^65–67^1
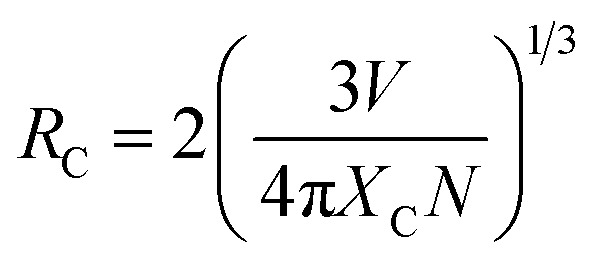
where, *V* (=916.439 Å^3^) is the volume of the unit cell, *X*_C_ = 1 mol%, and *N* = 4 number of cationic sites in the unit cell. The value of *R*_C_ for both rare earth ion-activated LiAl(PO_3_)_4_ phosphors was found to be 35.244. If the value of *R*_C_ is more than 5, the electric multipolar interaction will be the predominant mechanism causing the concentration quenching event, otherwise, an exchange interaction will be responsible. Because the calculated value of *R*_C_ is more than 5 it indicates that the electric multipolar interaction is responsible for the concentration quenching effect in rare earth ion-activated LiAl(PO_3_)_4_ phosphor.

**Fig. 8:**
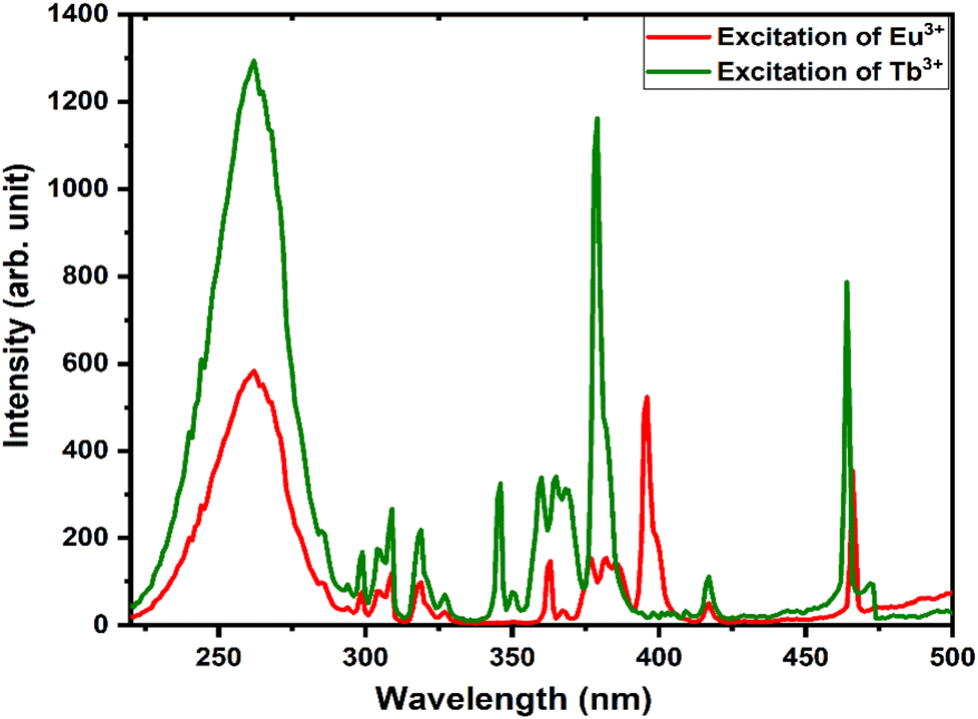
Spectral overlap between the excitations of Tb^3+^ and Eu^3+^ activated LiAl(PO_3_)_4_ phosphors.

The energy transfer criterion was satisfied by the spectral overlap shown in Fig. 8. The spectral overlapping occurred between the excitation–excitation of Tb^3+^ and Eu^3+^ ion-activated LiAl(PO_3_)_4_ phosphors. As per the criteria, the excitations of both rare earths overlapped, and it was observed that the excitation of Eu^3+^ is on the higher wavelength side as compared to the excitation of Tb^3+^ ions. Therefore, the energy can be transferred from Tb^3+^ to Eu^3+^ ions.

**Fig. 9:**
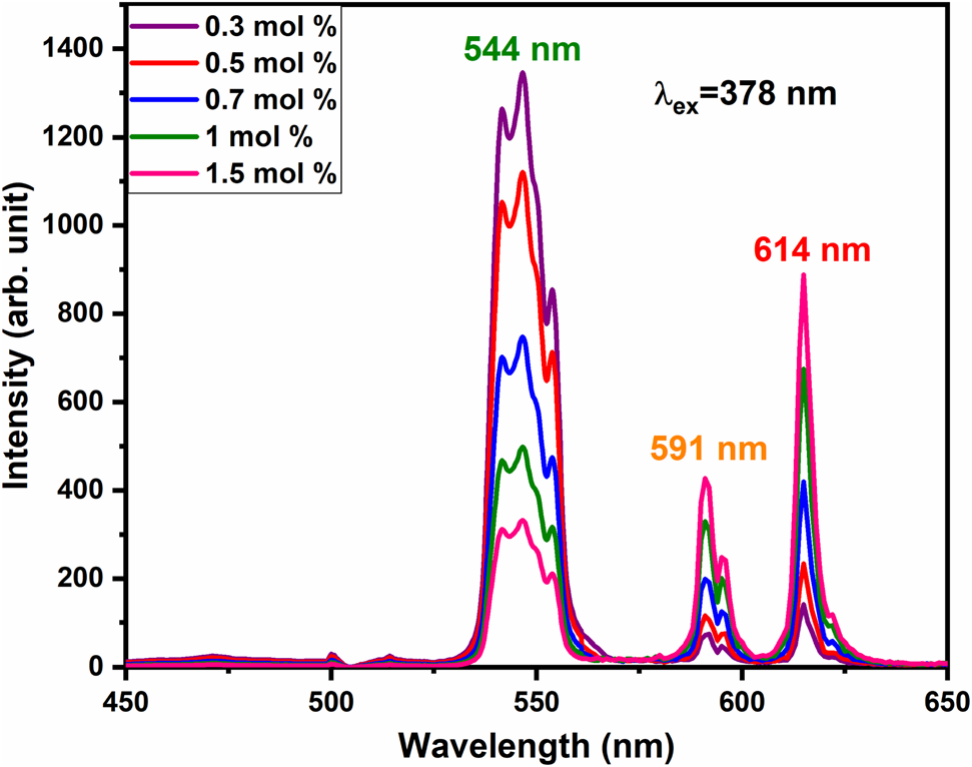
PL emission spectra of LiAl(PO_3_)_4_:Tb^3+^ (1 mol%), *y*Eu^3+^ phosphors at an excitation of 378 nm.

To satisfy the energy transfer criteria, Tb^3+^, Eu^3+^ co-activated LiAl(PO_3_)_4_ phosphors were prepared by keeping the concentration of Tb^3+^ ions constant at 1 mol% and Eu^3+^ concentration varying from 0.3 mol% to 1.5 mol%. The PL emission spectra of this co-activated phosphor triggered at 378 nm excitation are displayed in Fig. 9 and have three characteristic emission peaks centred at 544 nm 591 nm and 614 nm. The peaks attributed in emission spectra are already known for Tb^3+^ and Eu^3+^ ions and their transitions are already mentioned in the above section.

**Fig. 10:**
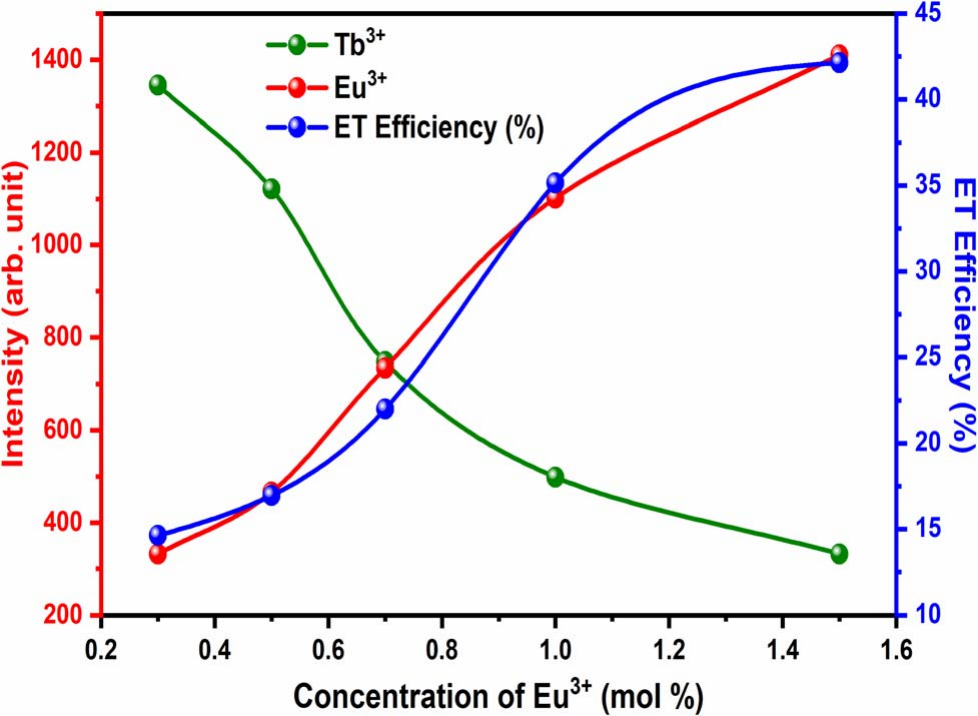
ET efficiency and PL emission intensity of Tb^3+^ and Eu^3+^ for different concentrations of Eu^3+^.

**Fig. 11:**
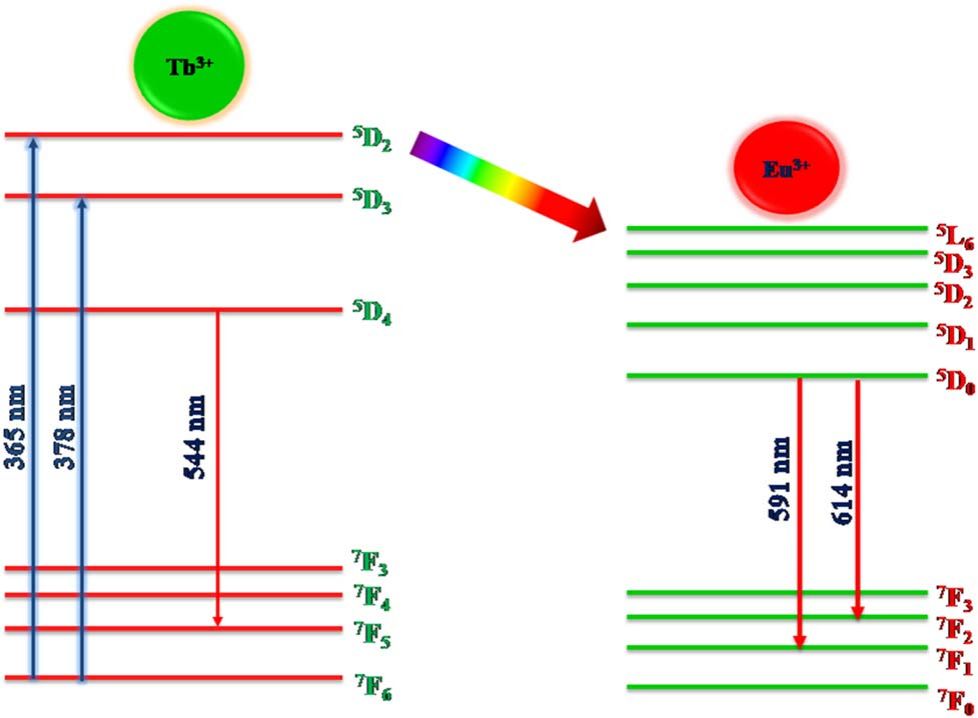
Energy-level diagram and energy transfer from Tb^3+^ to Eu^3+^.

The variation in the intensity of Tb^3+^ and Eu^3+^ emission peaks is depicted in Fig. 10. As observed, the intensity of the peak centred at 544 nm (Tb^3+^ ions) was diminished and peaks centred at 591 nm and 614 nm (Eu^3+^ ions) increased with the concentration of Eu^3+^ ions. This shows the energy is transferred from Tb^3+^ ions to Eu^3+^ ions. The energy transfer efficiency (*η*_T_) from Tb^3+^ to Eu^3+^ can be calculated using the following equation;^68^2
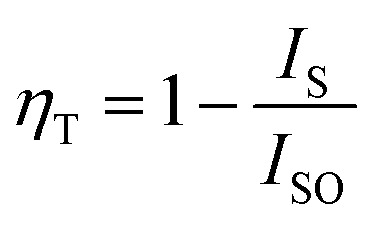
where *I*_S_ and *I*_SO_ are the luminescence intensities of Tb^3+^ with and without the presence of Eu^3+^, respectively. The *η*_T_ from Tb^3+^ to Eu^3+^ in LiAl(PO_3_)_4_ phosphors was calculated as a function of the Eu^3+^ concentration, displayed in Fig. 10. The value of *η*_T_ was found to increase gradually with an increase in Eu^3+^ content. When the Eu^3+^ ion concentration increased to 1.5 mol%, the transfer efficiency increased to 42.13%, indicating an efficient energy transfer from Tb^3+^ to Eu^3+^. The energy-level diagram of Tb^3+^ to Eu^3+^ ions showing a possible scheme of energy transfer is displayed in Fig. 11.

**Fig. 12:**
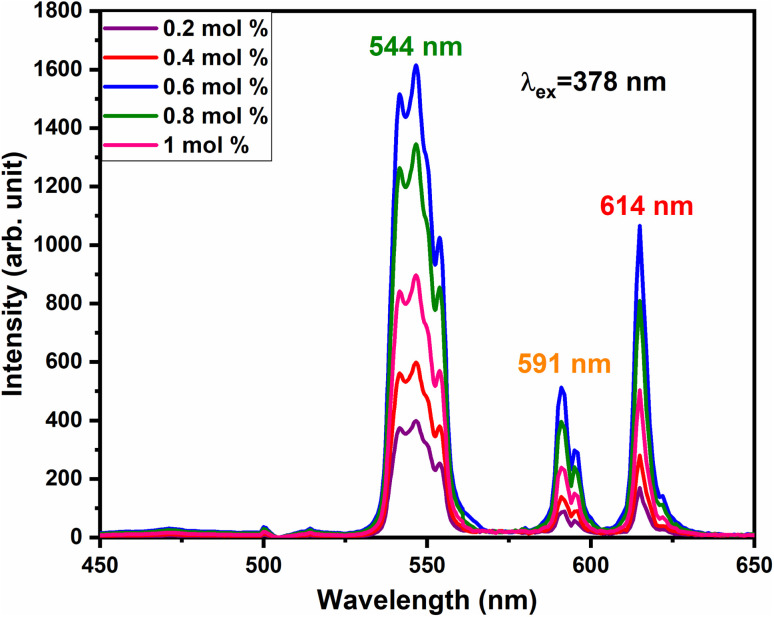
PL emission spectra of LiAl(PO_3_)_4_:Tb^3+^(1 mol%), Eu^3+^(1.5 mol%), zNa^+^ phosphors at 378 nm excitation.

**Fig. 13:**
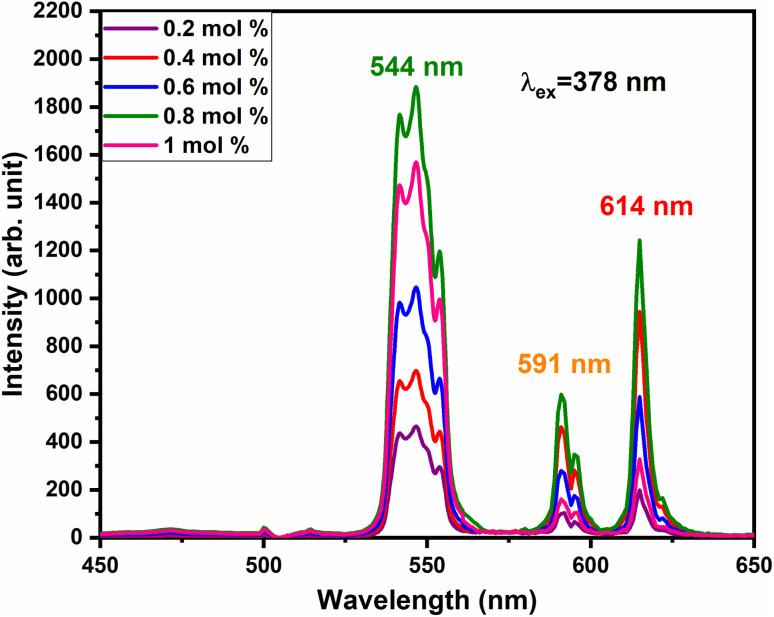
PL emission spectra of LiAl(PO_3_)_4_:Tb^3+^(1 mol%), Eu^3+^(1.5 mol%), zK^+^ phosphors at 378 nm excitation.

To investigate the charge compensation effect on Tb^3+^, Eu^3+^ co-activated LiAl(PO_3_)_4_ phosphors, alkali metal ions (Na^+^ and K^+^) were introduced as charge compensators. The charge compensation phenomena were observed in the LiAl(PO_3_)_4_:Tb^3+^–Eu^3+^ host lattice as rare earth ions (Tb^3+^/Eu^3+^) doped into the LiAl(PO_3_)_4_ phosphor. As per the Kröger–Vink notation,^69^ the charge compensation is required for 
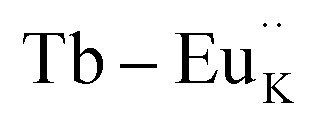
 sites.The proximity of charge compensating defects (almost certainly 
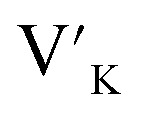
 vacancies or 
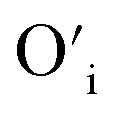
 interstitials) gives rise to local deformation of the EuO_4_ tetragonal and lifts the inversion symmetry and the charge compensation phenomena that occurred here. The PL emission spectra of Tb^3+^, Eu^3+^ co-activated LiAl(PO_3_)_4_ phosphors doped with Na^+^ and K^+^ as charge compensators are shown in Fig. 12 and 13, respectively. The charge compensation of Na^+^ and K^+^ ions make a significant impact on the PL intensity of Tb^3+^, Eu^3+^ co-activated LiAl(PO_3_)_4_ phosphors. The PL intensity was significantly enhanced by 1.2 and 1.4 times that of the Tb^3+^, Eu^3+^ co-activated LiAl(PO_3_)_4_ phosphors when charge compensators Na^+^ and K^+^ were introduced. To manifest the charge compensation effect of alkali metals the optimum intense sample in the co-doped sample was used. The PL emission spectra of 1 mol% Tb^3+^, 1.5 mol% Eu^3+^ co-activated LiAl(PO_3_)_4_ phosphors displayed in Fig. 12 and 13 by charge compensating Na^+^ and K^+^ consisted of different mol% of Na^+^ and K^+^ ions, respectively. It was observed that the PL emission intensity enhanced by both the charge compensation and the optimum sample obtained in both charge compensations is 0.6 mol% for Na^+^ and 0.8 mol% for K^+^ ions.

However, the ionic radius of Na^+^ ions is 1.18 Å and for K^+^ is 1.51 Å. Comparatively, the ionic radius of the Na^+^ ion is close to that of the Li^+^ ion. Thus, the doping of Na^+^ ion has a weaker strengthening effect on the crystal field and K^+^ doping has a stronger strengthening effect on the crystal field. Therefore, PL intensity was comparatively enhanced in K^+^ doping than in Na^+^ doping.

### 3.4 Photometric characterization

**Fig. 14:**
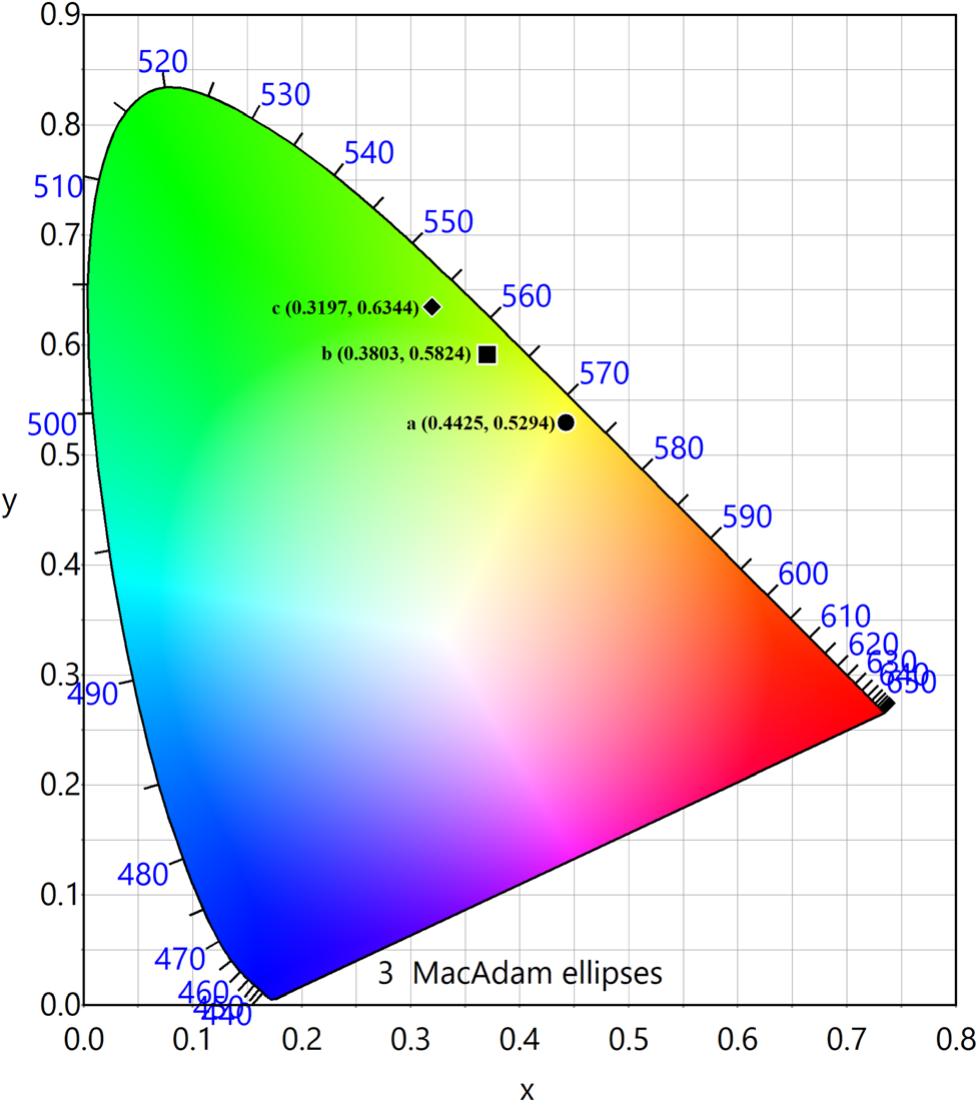
CIE chromaticity diagram of (a) LiAl(PO_3_)_4_: 1 mol% Tb^3+^, 1 mol% Eu^3+^, (b) LiAl(PO_3_)_4_: 1 mol% Tb^3+^, 1 mol% Eu^3+^, 0.6 mol% Na^+^ and (c) LiAl(PO_3_)_4_: 1 mol% Tb^3+^, 1 mol% Eu^3+^, and 0.8 mol% K^+^ phosphors.

The PL emission spectra of the produced Tb^3+^, Eu^3+^ co-activated LiAl(PO_3_)_4_ phosphors were examined using Commission de I Eclairage (CIE) coordinates, as shown in Fig. 14. The useful criteria to gauge the caliber of the luminescence phosphor are the CIE chromaticity coordinates. Table 2 displays the CIE coordinates. The PL emission was shifted towards the red region on charge compensating alkali metal ions into Tb^3+^, Eu^3+^ co-activated LiAl(PO_3_)_4_ phosphors with efficiency enhancement. This shows the photochromic nature of the prepared phosphors. As shown in Table 2, the produced phosphors' colour purity varied. The provided formula determines colour purity of the phosphor;^7,70,71^3
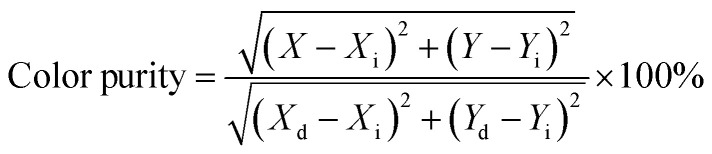
where, (*X*, *Y*), (*X*_i_, *Y*_i_), and (*X*_d_, *Y*_d_) are color-coordinates of the sample point and CIE equal-energy illuminant, and dominant wavelength of the light source, respectively.

**Table 2:** Overview of the CIE chromaticity and color purity of LiAl(PO_3_)_4_-doped phosphors

Sr no.	Sample	*X*	*Y*	*X*_d_	*Y*_d_	Color purity (%)
a	LiAl(PO_3_)_4_: 1 mol% Tb^3+^, 1 mol% Eu^3+^	0.4425	0.5294	0.3017	0.6884	54.36
b	LiAl(PO_3_)_4_: 1 mol% Tb^3+^, 1 mol% Eu^3+^, 0.6 mol% Na^+^	0.3803	0.5824	0.3017	0.6884	69.47
c	LiAl(PO_3_)_4_: 1 mol% Tb^3+^, 1 mol% Eu^3+^, 0.8 mol% K^+^	0.3197	0.6344	0.3017	0.6884	66.07

## 4. Conclusion

The charge compensation effect and photochromic properties of LiAl(PO_3_)_4_: *x* mol% Tb^3+^, *y* mol% Eu^3+^, *z* mol% R^+^ (R = Na, K) doped materials were investigated in this work. LiAl(PO_3_)_4_: *x* mol% Tb^3+^, *y* mol% Eu^3+^, *z* mol% R^+^ (R = Na, K) phosphors were prepared by the wet chemical method. The crystal structure of the prepared sample was in the orthorhombic crystal system. The surface morphological behavior and particle size of the prepared phosphor are in the sub-micrometer region, as confirmed by SEM analysis. In the green to red regions, the PL examination of this phosphor revealed four significant emission peaks. In this phosphor, the efficiency of the mechanism of energy transfer from Tb^3+^ to Eu^3+^ ions was determined to be 42.13%. Moreover, the charge compensation of the alkali metals can help in improving the intensity of the prepared phosphors by a factor of approximately 1.3 and also shows the photochromic nature of the phosphor. Therefore, all of the indicated results support the studied phosphor material for standing in the race for the development of efficient phosphors for use in color-tunable WLEDs and displays.

## Author contributions

Prashant N. Parale – investigation, formal analysis, writing – original draft. Abhijeet R. Kadam – visualization, methodology, investigation, validation, writing – original draft. S. J. Dhoble – supervision, visualization, conceptualization, project administration. K.V. Dabre – supervision, visualization, conceptualization, writing – original draft.

## Conflicts of interest

There are no conflicts to declare.

## Supplementary Material
